# The genome sequence of the spiny starfish, *Marthasterias glacialis* (Linnaeus, 1758)

**DOI:** 10.12688/wellcomeopenres.17344.1

**Published:** 2021-11-05

**Authors:** Mara K.N. Lawniczak, Darwin Tree of Life Barcoding collective, Wellcome Sanger Institute Tree of Life programme, Wellcome Sanger Institute Scientific Operations: DNA Pipelines collective, Tree of Life Core Informatics collective, Darwin Tree of Life Consortium

**Affiliations:** 1 Tree of Life, Wellcome Sanger Institute, Cambridge, UK

**Keywords:** Marthasterias glacialis, spiny starfish, genome sequence, chromosomal

## Abstract

We present a genome assembly from an individual *Marthasterias glacialis *(the spiny starfish; Echinodermata; Asteroidea; Forcipulatida; Asteriidae). The genome sequence is 521 megabases in span. The majority of the assembly, 99.44%, is scaffolded into 22 chromosomal pseudomolecules. The mitochondrial genome has also been assembled, and is 16 kb in span.

## Species taxonomy

Eukaryota; Metazoa; Echinodermata; Eleutherozoa; Asterozoa; Asteroidea; Forcipulatacea; Forcipulatida; Asteriidae; Marthasterias; *Marthasterias glacialis* (Linnaeus, 1758) (NCBI:txid7609).

## Background

**Figure 1.**
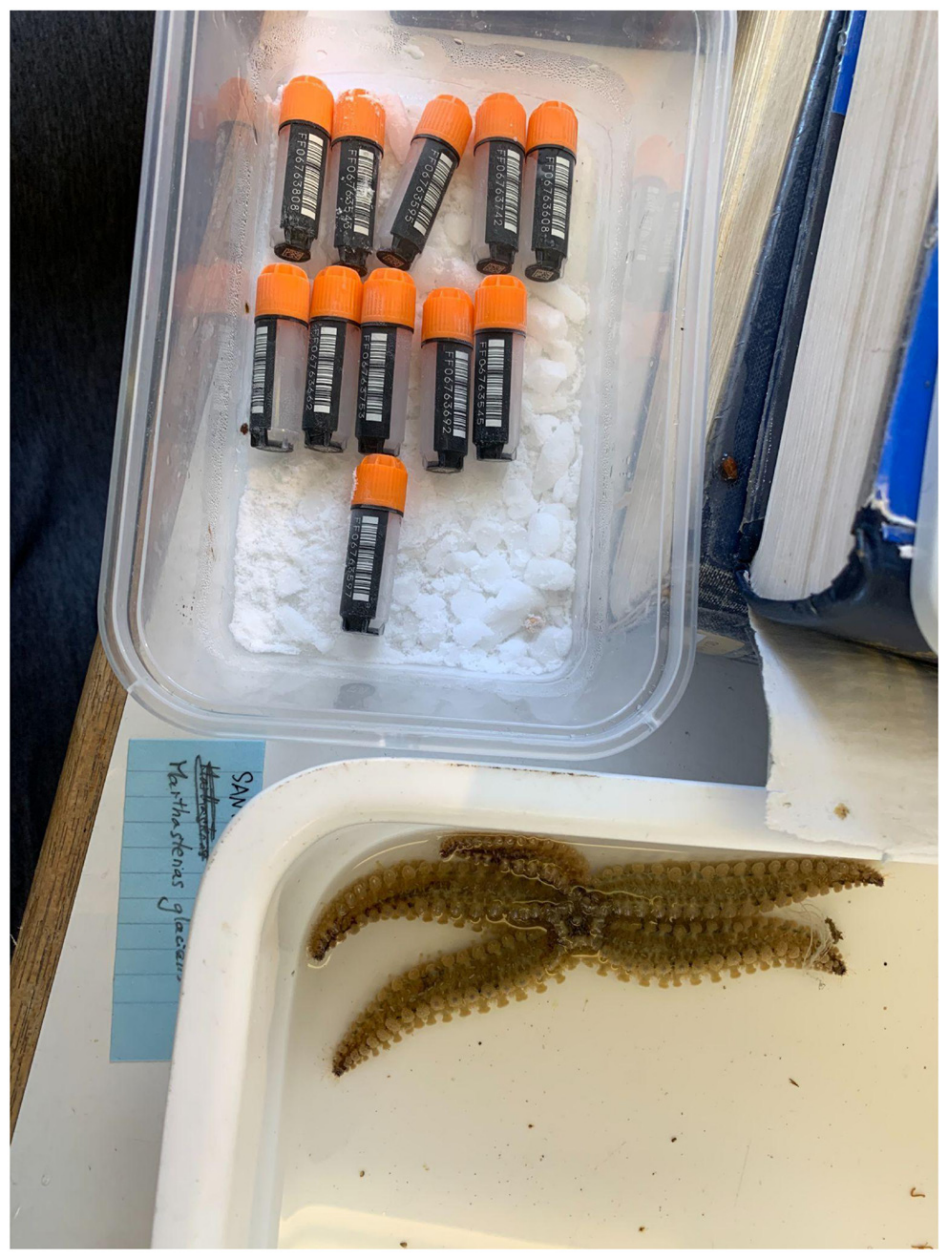
An image of the sequenced specimen, eaMarGlac1, taken immediately prior to processing and preservation.

The spiny starfish, *Marthasterias glacialis*, is an opportunistic and generalist feeder distributed widely throughout Europe ( https://www.sealifebase.ca/summary/Marthasterias). In England and Scotland, it is only found on the west coast, and the specimen sequenced here was captured on the Isle of Cumbrae in Scotland. It was found at a depth of about 4 metres on the northeast of the island, and processed in the lab of FSC Millport in August 2020. An image of the sequenced specimen just prior to processing is provided in Figure 1. Cytochrome Oxidase I studies of the species throughout Europe have shown that there are two divergent lineages in the Mediterranean ( Pérez-Portela *et al*., 2010). This new reference genome will assist in better understanding population structure within the species across its full range.

## Genome sequence report

The genome was sequenced from a single *M. glacialis* of unknown sex collected from Farland Point, Great Cumbrae, North Ayrshire, Scotland (latitude 55.746815, longitude -4.914907). A total of 49-fold coverage in Pacific Biosciences single-molecule long reads and 69-fold coverage in 10X Genomics read clouds were generated. Primary assembly contigs were scaffolded with chromosome conformation Hi-C data. Manual assembly curation corrected 363 missing/misjoins and removed 42 haplotypic duplications, reducing the assembly size by 2.74% and scaffold number by 61.31%, and increasing the scaffold N50 by 37.41%.

**Figure 2.**
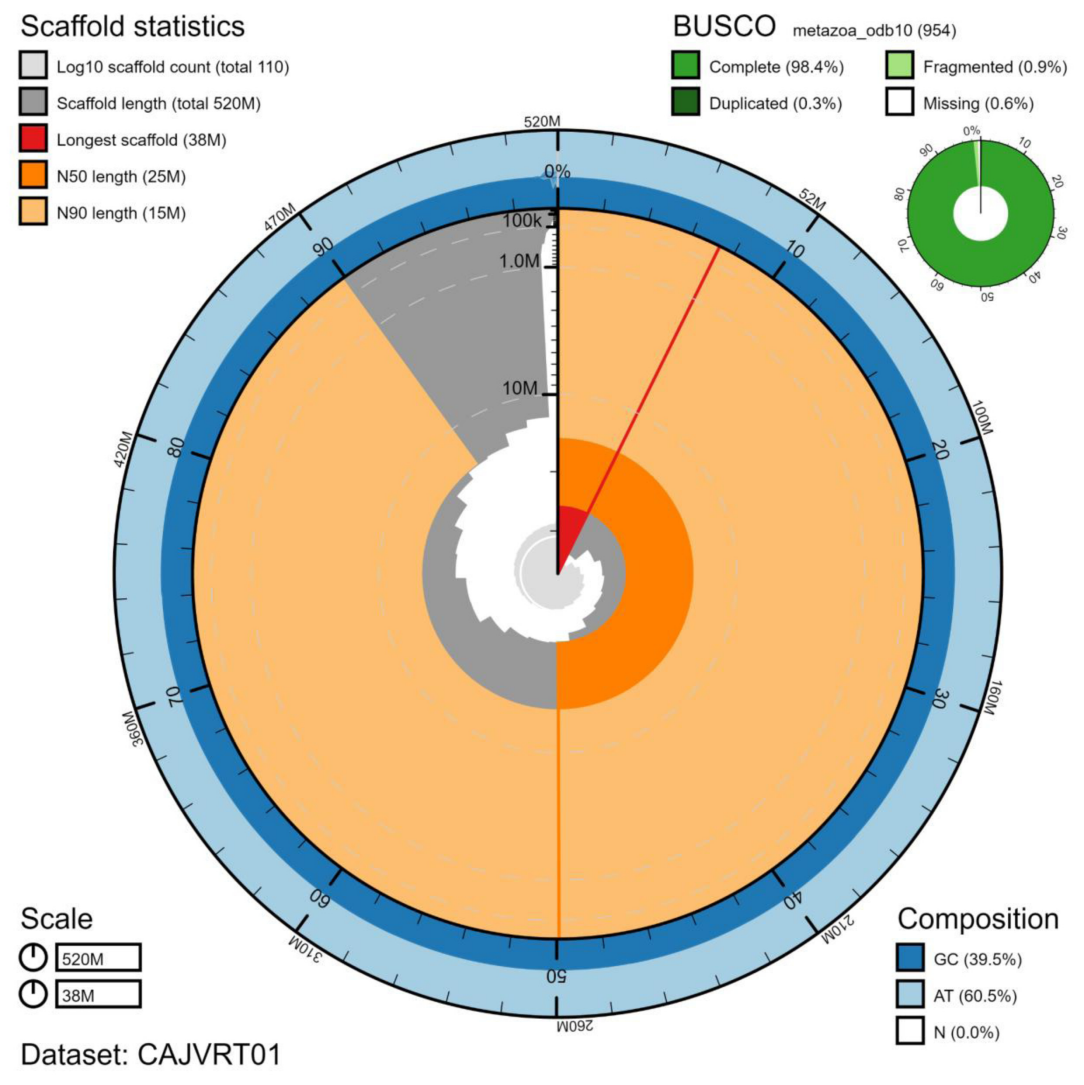
Genome assembly of *Marthasterias glacialis*, eaMarGlac1.1: metrics. The BlobToolKit Snailplot shows N50 metrics and BUSCO gene completeness. The main plot is divided into 1,000 size-ordered bins around the circumference with each bin representing 0.1% of the 520,959,849 bp assembly. The distribution of scaffold lengths is shown in dark grey with the plot radius scaled to the longest scaffold present in the assembly (38,475,774 bp, shown in red). Orange and pale-orange arcs show the N50 and N90 scaffold lengths (25,218,880 and 15,203,031 bp), respectively. The pale grey spiral shows the cumulative scaffold count on a log scale with white scale lines showing successive orders of magnitude. The blue and pale-blue area around the outside of the plot shows the distribution of GC, AT and N percentages in the same bins as the inner plot. A summary of complete, fragmented, duplicated and missing BUSCO genes in the metazoa_odb10 set is shown in the top right. An interactive version of this figure is available at https://blobtoolkit.genomehubs.org/view/eaMarGlac1.1/dataset/CAJVRT01/snail.

The final assembly has a total length of 521 Mb in 106 sequence scaffolds with a scaffold N50 of 25 Mb ( Table 1). Of the assembly sequence, 99.44% was assigned to 22 chromosomal-level scaffolds, representing 22 autosomes (numbered by sequence length) ( Figure 2– Figure 5; Table 2). The assembly has a BUSCO ( Simão *et al.*, 2015) v5.1.2 completeness of 98.4% using the metazoa_odb10 reference set. While not fully phased, the assembly deposited is of one haplotype. Contigs corresponding to the second haplotype have also been deposited.

**Table 1.** Genome data for *Marthasterias glacialis*, eaMarGlac1.1.

*Project accession data*	
Assembly identifier	eaMarGlac1.1
Species	*Marthasterias glacialis*
Specimen	eaMarGlac1
NCBI taxonomy ID	7609
BioProject	PRJEB45116
BioSample ID	SAMEA7522991
Isolate information	Unknown sex; legs
*Raw data accessions*	
PacificBiosciences SEQUEL II	ERR6436374
10X Genomics Illumina	ERR6054755-ERR6054758
Hi-C Illumina	ERR6054759
Illumina polyA RNA-Seq	ERR6054760
*Genome assembly*	
Assembly accession	GCA_911728455.1
*Accession of alternate haplotype*	GCA_911728445.1
Span (Mb)	521
Number of contigs	574
Contig N50 length (Mb)	1.9
Number of scaffolds	106
Scaffold N50 length (Mb)	25.2
Longest scaffold (Mb)	38.5
BUSCO ^*^ genome score	C:98.4%[S:98.1%,D:0.3%], F:0.9%,M:0.6%,n:954

*BUSCO scores based on the metazoa_odb10 BUSCO set using v5.1.2. C= complete [S= single copy, D=duplicated], F=fragmented, M=missing, n=number of orthologues in comparison. A full set of BUSCO scores is available at https://blobtoolkit.genomehubs.org/view/eaMarGlac1.1/dataset/CAJVRT01/busco.

**Figure 3.**
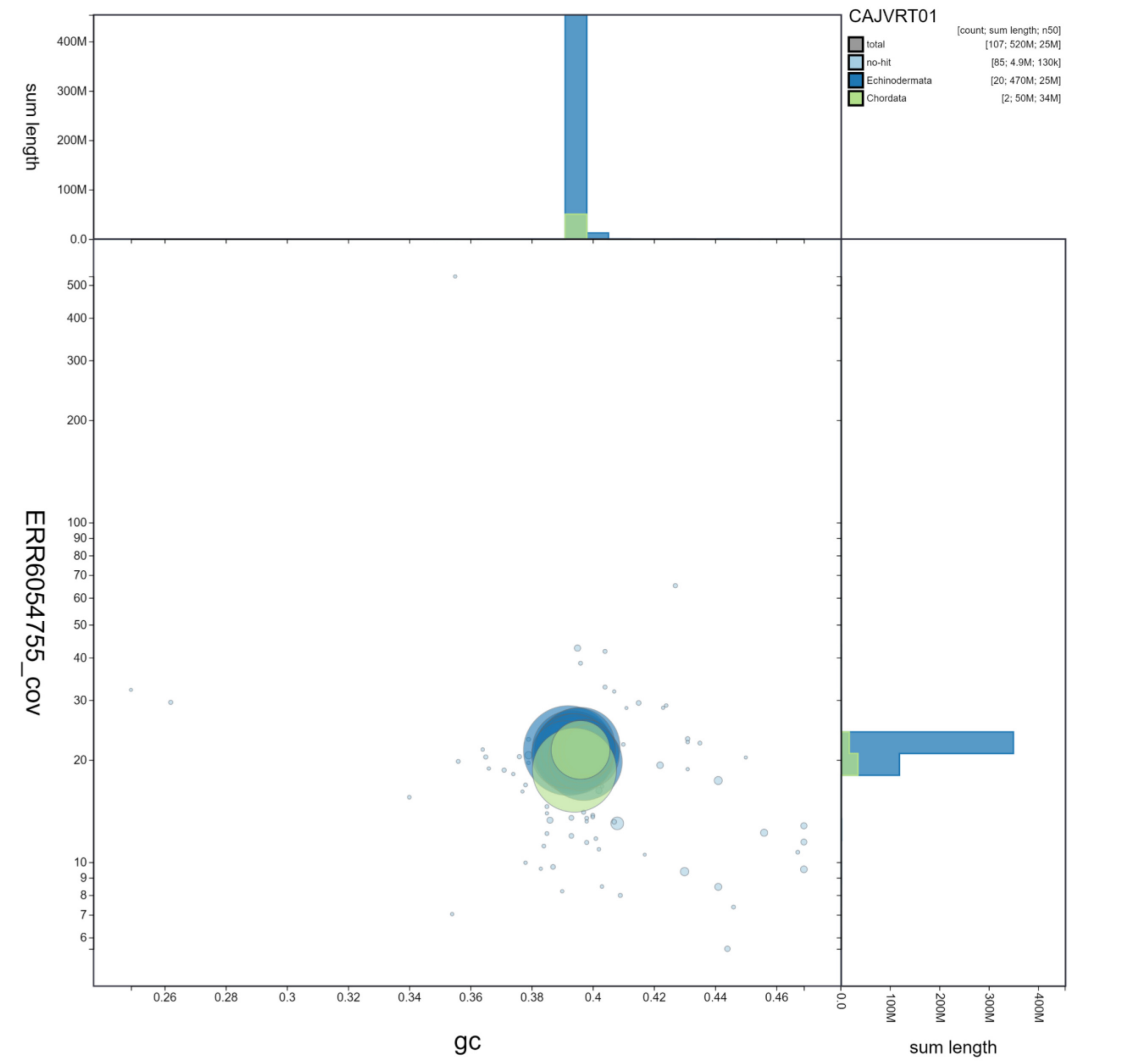
Genome assembly of *Marthasterias glacialis*, eaMarGlac1.1: GC-coverage. BlobToolKit GC-coverage plot. Scaffolds are coloured by phylum. Circles are sized in proportion to scaffold length. Histograms show the distribution of scaffold length sum along each axis. Scaffolds labelled Chordata are spurious and assumed to reflect some confusion in the sequence databases from which data is pulled. An interactive version of this figure is available at https://blobtoolkit.genomehubs.org/view/eaMarGlac1.1/dataset/CAJVRT01/blob.

**Figure 4.**
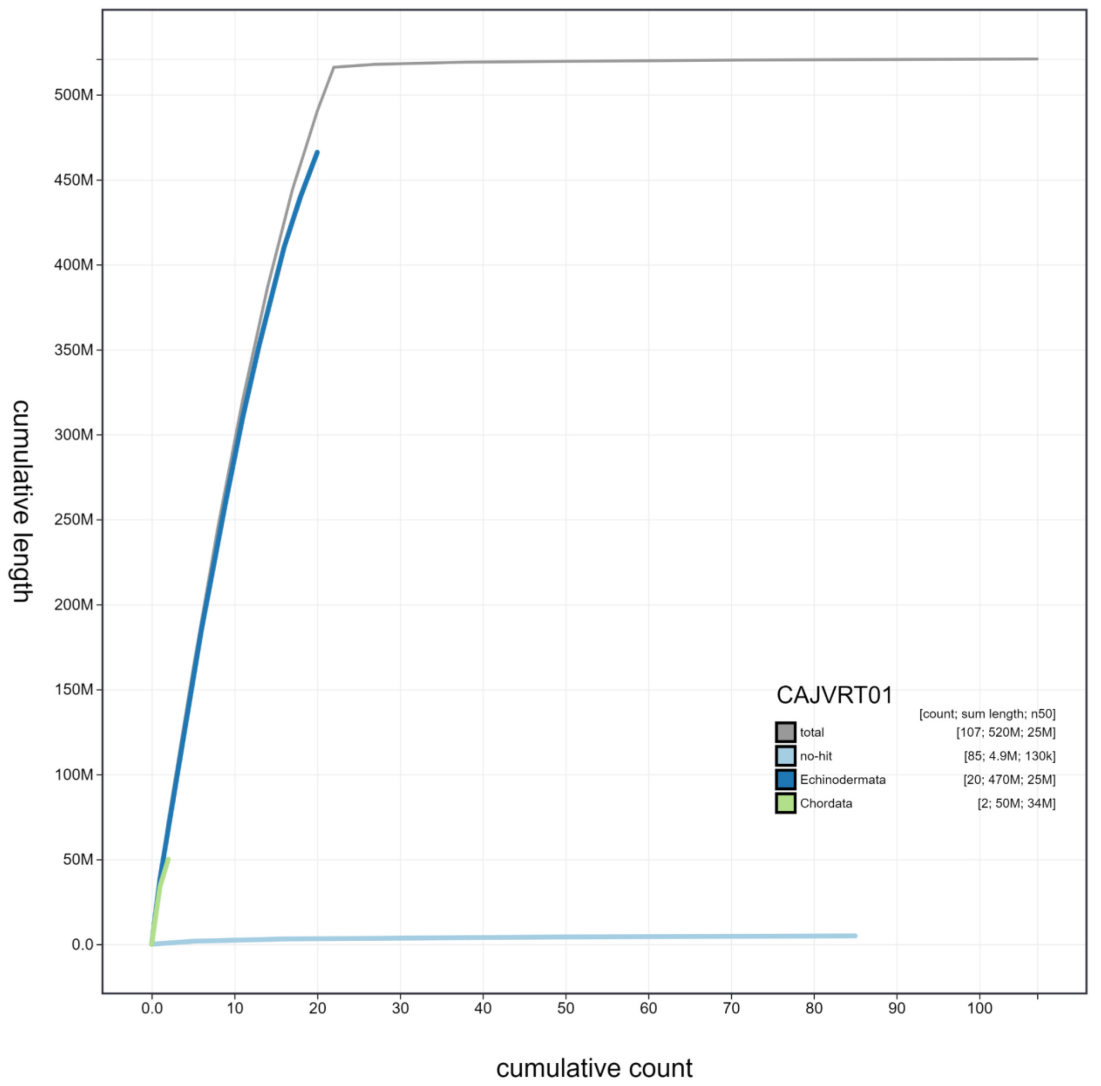
Genome assembly of *Marthasterias glacialis*, eaMarGlac1.1: cumulative sequence. BlobToolKit cumulative sequence plot. The grey line shows cumulative length for all scaffolds. Coloured lines show cumulative lengths of scaffolds assigned to each phylum using the buscogenes taxrule. An interactive version of this figure is available at https://blobtoolkit.genomehubs.org/view/eaMarGlac1.1/dataset/CAJVRT01/cumulative.

**Figure 5.**
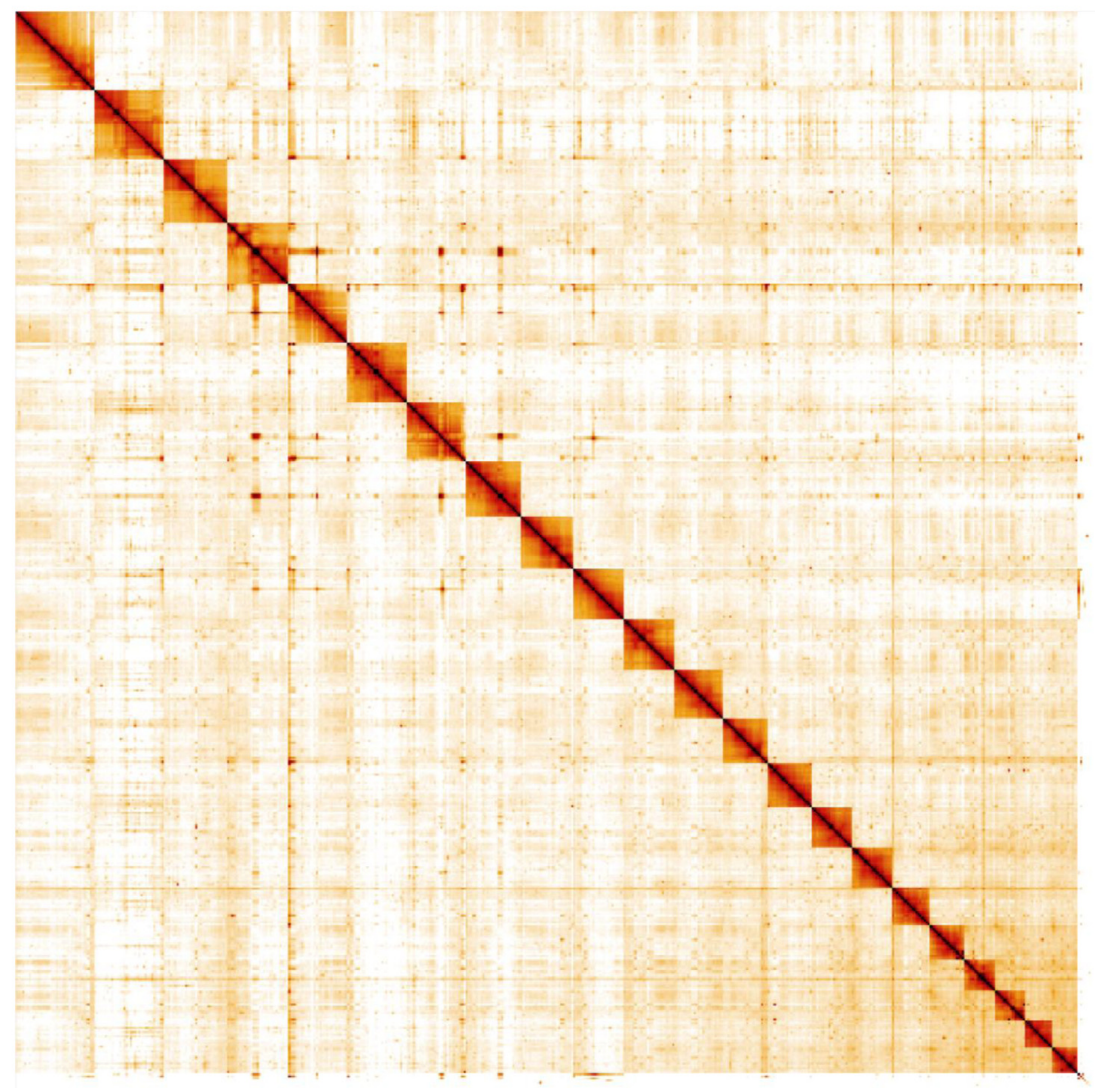
Genome assembly of *Marthasterias glacialis*, eaMarGlac1.1: Hi-C contact map. Hi-C contact map of the eaMarGlac1.1 assembly, visualised in HiGlass. Chromosomes are shown in order of size from left to right and top to bottom.

**Table 2.** Chromosomal pseudomolecules in the genome assembly of *Marthasterias glacialis*, eaMarGlac1.1.

INSDC accession	Chromosome	Size (Mb)	GC%
OU452219.1	1	38.48	39.2
OU452220.1	2	33.79	39.4
OU452221.1	3	30.68	39.4
OU452222.1	4	29.52	39.6
OU452223.1	5	29.13	39.3
OU452224.1	6	28.78	39.4
OU452225.1	7	28.21	39.7
OU452226.1	8	26.68	39.5
OU452227.1	9	25.22	39.3
OU452228.1	10	25.09	39.3
OU452229.1	11	24.75	39.5
OU452230.1	12	23.37	39.5
OU452231.1	13	21.44	39.4
OU452232.1	14	21.39	39.1
OU452233.1	15	19.79	39.8
OU452234.1	16	19.49	39.6
OU452235.1	17	18.12	39.6
OU452236.1	18	16.22	39.6
OU452237.1	19	15.20	39.7
OU452238.1	20	14.95	39.8
OU452239.1	21	13.34	39.8
OU452240.1	22	12.43	39.9
OU452241.1	MT	0.02	35.6
-	Unplaced	4.86	41

## Methods

### Sample acquisition and nucleic acid extraction

A single *M. glacialis* of unknown sex was collected from Farland Point, Great Cumbrae, North Ayrshire, Scotland (latitude 55.746815, longitude -4.914907) by Mara Lawniczak (Wellcome Sanger Institute (hereafter *Sanger*)). The specimen was collected by hand while snorkelling, and identified by Richard Durbin (University of Cambridge/Sanger) and Mark Blaxter (Sanger) and preserved and processed on dry ice by Mara Lawniczak.

DNA was extracted at the Tree of Life laboratory, WSI. The *M. glacialis* sample was weighed and dissected on dry ice with tissue set aside for RNA extraction and Hi-C sequencing. Leg tissue was cryogenically disrupted to a fine powder using a Covaris cryoPREP Automated Dry Pulveriser, receiving multiple impacts. Fragment size analysis of 0.01-0.5 ng of DNA was then performed using an Agilent FemtoPulse. High molecular weight (HMW) DNA was extracted using the Qiagen MagAttract HMW DNA extraction kit. Low molecular weight DNA was removed from a 200-ng aliquot of extracted DNA using 0.8X AMpure XP purification kit prior to 10X Chromium sequencing; a minimum of 50 ng DNA was submitted for 10X sequencing. HMW DNA was sheared into an average fragment size between 12–20 kb in a Megaruptor 3 system with speed setting 30. Sheared DNA was purified by solid-phase reversible immobilisation using AMPure PB beads with a 1.8X ratio of beads to sample to remove the shorter fragments and concentrate the DNA sample. The concentration of the sheared and purified DNA was assessed using a Nanodrop spectrophotometer and Qubit Fluorometer and Qubit dsDNA High Sensitivity Assay kit. Fragment size distribution was evaluated by running the sample on the FemtoPulse system.

RNA was extracted from further leg tissue in the Tree of Life Laboratory at the WSI using TRIzol (Invitrogen), according to the manufacturer’s instructions. RNA was then eluted in 50 μl RNAse-free water and its concentration assessed using a Nanodrop spectrophotometer and Qubit Fluorometer using the Qubit RNA Broad-Range (BR) Assay kit. Analysis of the integrity of the RNA was done using Agilent RNA 6000 Pico Kit and Eukaryotic Total RNA assay.

### Sequencing

Pacific Biosciences HiFi circular consensus and 10X Genomics Chromium read cloud sequencing libraries were constructed according to the manufacturers’ instructions. Poly(A) RNA-Seq libraries were constructed using the NEB Ultra II RNA Library Prep kit. Sequencing was performed by the Scientific Operations core at Sanger on Pacific Biosciences SEQUEL II (HiFi), Illumina HiSeq X (10X) and Illumina HiSeq 4000 (RNA-Seq) instruments. Hi-C data were generated using the Arima v2 Hi-C kit and sequenced on an Illumina NovaSeq 6000 instrument.

### Genome assembly

Assembly was carried out with HiCanu ( Nurk *et al.*, 2020). Haplotypic duplication was identified and removed with purge_dups (with purging in the middle of contigs) ( Guan *et al.*, 2020). Scaffolding with Hi-C data ( Rao *et al.*, 2014) was carried out with SALSA2 ( Ghurye *et al.*, 2019). The Hi-C scaffolded assembly was polished with the 10X Genomics Illumina data by aligning to the assembly with longranger align, calling variants with freebayes ( Garrison & Marth, 2012). One round of the Illumina polishing was applied. The assembly was checked for contamination and corrected using the gEVAL system ( Chow *et al.*, 2016) as described previously ( Howe *et al.*, 2021). Manual curation was performed using gEVAL, HiGlass ( Kerpedjiev *et al.*, 2018) and Pretext. Regions of concern were identified and resolved using 10X longranger and genetic mapping data. The genome was analysed within the BlobToolKit environment ( Challis *et al.*, 2020). Table 3 contains a list of all software tool versions used, where appropriate.

**Table 3.** Software tools used.

Software tool	Version	Source
HiCanu	2.1	Nurk *et al.*, 2020
purge_dups	1.2.3	Guan *et al.*, 2020
longranger	2.2.2	https://support.10xgenomics.com/genome-exome/ software/pipelines/latest/advanced/other-pipelines
freebayes	1.3.1-17-gaa2ace8	Garrison & Marth, 2012
SALSA2	2.2	Ghurye *et al.*, 2019
MitoHiFi	1.0	Uliano-Silva *et al.*, 2021
gEVAL	N/A	Chow *et al.*, 2016
HiGlass	1.11.6	Kerpedjiev *et al.*, 2018
PretextView	0.1.x	https://github.com/wtsi-hpag/PretextView
BlobToolKit	2.6.2	Challis *et al.*, 2020

### Ethical/compliance issues

The materials that have contributed to this genome note were supplied by a Tree of Life collaborator. The Wellcome Sanger Institute employs a process whereby due diligence is carried out proportionate to the nature of the materials themselves, and the circumstances under which they have been/are to be collected and provided for use. The purpose of this is to address and mitigate any potential legal and/or ethical implications of receipt and use of the materials as part of the research project, and to ensure that in doing so we align with best practice wherever possible.

The overarching areas of consideration are:

- Ethical review of provenance and sourcing of the material;
- Legality of collection, transfer and use (national and international).

Each transfer of samples is undertaken according to a Research Collaboration Agreement or Material Transfer Agreement entered into by the Tree of Life collaborator, Genome Research Limited (operating as the Wellcome Sanger Institute) and in some circumstances other Tree of Life collaborators.

## Data availability

European Nucleotide Archive: Marthasterias glacialis (spiny starfish). Accession number PRJEB45116; https://identifiers.org/ena.embl/PRJEB45116.

The genome sequence is released openly for reuse. The *M. glacialis* genome sequencing initiative is part of the Darwin Tree of Life (DToL) project. All raw sequence data and the assembly have been deposited in INSDC databases. The genome will be annotated using the RNA-Seq data and presented through the Ensembl pipeline at the European Bioinformatics Institute. Raw data and assembly accession identifiers are reported in Table 1.

## Author information

Members of the Darwin Tree of Life Barcoding collective are listed here: https://doi.org/10.5281/zenodo.4893704.

Members of the Wellcome Sanger Institute Tree of Life programme collective are listed here: https://doi.org/10.5281/zenodo.5377053.

Members of Wellcome Sanger Institute Scientific Operations: DNA Pipelines collective are listed here: https://doi.org/10.5281/zenodo.4790456.

Members of the Tree of Life Core Informatics collective are listed here: https://doi.org/10.5281/zenodo.5013542.

Members of the Darwin Tree of Life Consortium are listed here: https://doi.org/10.5281/zenodo.4783559.
