# Supplementary material for: The genome sequence of the spiny starfish, Marthasterias glacialis (Linnaeus, 1758)
Source: Wellcome Open Res. 2021 Nov 5;6:295. [Version 1] doi: 10.12688/wellcomeopenres.17344.1 (PMC10015122; doi:10.12688/wellcomeopenres.17344.1)
Supplement: Peer review history

# Peer review: Reviewer response for version 1

**DOI:** 10.21956/wellcomeopenres.19174.r55125

**Published:** 2023-03-14

**Authors:** Shunsuke Yaguchi

**Affiliations:** 1 Shimoda Marine Research Center, Tsukuba Daigaku, Tsukuba, Ibaraki Prefecture, Japan

The manuscript describes the whole genome assembly of *M. glacialis*. The authors detail the sampling, reading, and assembly process of the genome. Thanks to recent advancements in sequencing and bioinformatics technologies, it has become much easier to read genomes of many non-model organisms compared to the previous decade. However, not many journals accept simple "read and assembled" genome papers like this manuscript. Therefore, I'm personally pleased to see this Data Note on genome reading in this journal.

 Minor points; 

1. Why didn't the authors determine the specimen's sex?
2. Which part of the leg (generally referred to as the arm by other reviewers) did the authors use for sampling? As the leg or arm is an organ and not a tissue, the authors need to indicate whether they used the whole leg (arm) or just a part of it.
3. The scale of Figure 2 (520M/38M) may be a mistake.

Are sufficient details of methods and materials provided to allow replication by others?

Yes

Is the rationale for creating the dataset(s) clearly described?

Yes

Are the datasets clearly presented in a useable and accessible format?

Yes

Are the protocols appropriate and is the work technically sound?

Yes

Reviewer Expertise:

Developmental biology and physiology of echinoderm embryos and larvae.

I confirm that I have read this submission and believe that I have an appropriate level of expertise to confirm that it is of an acceptable scientific standard.

# Peer review: Reviewer response for version 1

**DOI:** 10.21956/wellcomeopenres.19174.r55122

**Published:** 2023-03-03

**Authors:** Charles A Ettensohn

**Affiliations:** 1 Carnegie Mellon University, Pittsburgh, Pennsylvania, USA

This data note describes a genome assembly of an unsexed individual of the spiny starfish ( *M. glacialis*). This work is part of the Darwin Tree of Life Project. The report is quite brief but the level of detail seems adequate and the assembly is publicly available. It has not been annotated and so will be of limited use to the community until that is completed. Certainly the annotated genome will be of interest to biologists of many stripes. 

**Minor questions:**

1. Why was the individual not sexed? It would be trivial to sample a gonad and determine the sex, assuming the gonad had at at least some mature gametes.
2. I have never heard reference to the "legs" of a seastar. Do you mean the arms? 

Are sufficient details of methods and materials provided to allow replication by others?

Yes

Is the rationale for creating the dataset(s) clearly described?

Yes

Are the datasets clearly presented in a useable and accessible format?

Yes

Are the protocols appropriate and is the work technically sound?

Yes

Reviewer Expertise:

Developmental biology and genomics, using echinoderms as model organisms

I confirm that I have read this submission and believe that I have an appropriate level of expertise to confirm that it is of an acceptable scientific standard.

# Peer review: Reviewer response for version 1

**DOI:** 10.21956/wellcomeopenres.19174.r47069

**Published:** 2021-12-15

**Authors:** Maurice R. Elphick

**Affiliations:** 1 School of Biological & Behavioural Sciences, Queen Mary University of London, London, UK

In this article the authors report the sequencing of the genome of the spiny starfish *Marthasterias glacialis*. This is the second starfish species to have its genome sequenced by the Wellcome Sanger Institute – a chromosomal assembly of the genome sequence of the common starfish *Asterias rubens* was submitted to NCBI on 12 February 2020 (PRJEB33974). This is relevant from a taxonomic perspective because both *Marthasterias glacialis* and *Asterias rubens* are species that belong to the same order (Forcipulatida) and family (Asteriidae). In contrast, the first starfish species to have its genome sequence reported, *Acanthaster planci* (Hall *et al.*, 2017 ^1^), belongs to a different order (Valvatida). It is to be expected, therefore, that the *A. rubens* and *M. glacialis* genomes will share more similarities than either genome shares with the genome of *A. planci*.

 Assembly of the *M. glacialis* genome sequence is consistent with a karyotype comprising 22 chromosomes, which is consistent with the karyotype of *A. rubens* based on the assembled genome sequence (PRJEB33974) and the karyotype of several starfish species, including *Asterias amurensis*, which were determined by Saotome and Komatsu in 2002 (Saotome and Komatsu, 2002 ^2^).

 The determination and public availability of the genome sequence of *Marthasterias glacialis *provides a superb resource for research on this species and starfish in general. Although *M. glacialis* is less widely distributed in UK waters than *A. rubens*, it has been used for experimental research. For example, the first detailed description of the anatomy of the starfish nervous system was largely based on analysis of specimens of *M. glacialis*, as reported by J.E. Smith in 1937 (Smith, 1937 ^3^). More recently, Yun *et al. *(2007 ^4^) reported the identification of four SALMFamide-type neuropeptides isolated from the radial nerve cords of *M. glacialis*.. With the availability of the genome sequence of *M. glacialis* there now exist exciting opportunities for molecular level investigations of many aspects of the biology of *M. glacialis* and comparison with *A. rubens* and other starfish species.

 This article provides a detailed description of the methods employed for sequencing and assembly of the *M. glacialis* genome and links to sequence data. I recommend the following corrections/amendments to the article:

1. Throughout the article the authors refer to the “legs” of the starfish as the source of the DNA used for genome sequencing. However, the term “leg” not is used customarily. The correct term is “arm(s)” or “ray(s)”.
2. In the legend of Figure 3 the authors state “Scaffolds labelled Chordata are spurious and assumed to reflect some confusion in the sequence databases from which data is pulled.” I suggest that this issue should be resolved.
3. The name *Marthasterias glacialis* should be in italics throughout the article and in the reference list “ *Marthasterias Glacialis*” should be changed to “ *Marthasterias glacialis*” in the cited article by Pérez-Portela *et al. *(2010).
4. The article states that the specimen analysed was of unknown sex. Were some of the arms preserved in fixative? It may be possible determine the sex of the specimen by histological analysis of the gonads, which are located at the base of the arms.
5. I don’t understand the scale on the bottom left of Figure 2. The same symbol represents different lengths of DNA (520 M and 38 M).

Are sufficient details of methods and materials provided to allow replication by others?

Partly

Is the rationale for creating the dataset(s) clearly described?

Partly

Are the datasets clearly presented in a useable and accessible format?

Yes

Are the protocols appropriate and is the work technically sound?

Yes

Reviewer Expertise:

I have expertise on starfish biology and analysis of genome sequence data. I do not have expertise on the technical details of genome sequencing and assembly.

I confirm that I have read this submission and believe that I have an appropriate level of expertise to confirm that it is of an acceptable scientific standard, however I have significant reservations, as outlined above.

## References

[1] : The crown-of-thorns starfish genome as a guide for biocontrol of this coral reef pest. *Nature* .2017;544(7649) : 10.1038/nature22033 231-234 10.1038/nature22033 28379940

[2] : Chromosomes of Japanese starfishes. *Zoolog Sci* .2002;19(10) : 10.2108/zsj.19.1095 1095-103 10.2108/zsj.19.1095 12426471

[3] : II - On the nervous system of the starfish Mathasterias glacialis (L.). *Phil. Trans. R. Soc. Lond.* .1937;227(542) : 10.1098/rstb.1937.0002 111-173 10.1098/rstb.1937.0002 Reference source

[4] : Identification of novel SALMFamide neuropeptides in the starfish Marthasterias glacialis. *Comp Biochem Physiol A Mol Integr Physiol* .2007;147(2) : 10.1016/j.cbpa.2007.02.002 536-42 10.1016/j.cbpa.2007.02.002 17337223
